# The SoCAP (Social Communication, Affiliation, and Presence) Taxonomy of Social Features: Scoping Review of Commercially Available eHealth Apps

**DOI:** 10.2196/49714

**Published:** 2024-09-03

**Authors:** Ian Kwok, Melanie Freedman, Lisa Kamsickas, Emily G Lattie, Dershung Yang, Judith Tedlie Moskowitz

**Affiliations:** 1 Feinberg School of Medicine Northwestern University Chicago, IL United States; 2 Dana Incorporated Novi, MI United States; 3 BrightOutcome Inc Buffalo Grove, IL United States

**Keywords:** eHealth, digital interventions, social features, taxonomy, computer-human interaction, social connection, engagement, eHealth apps, intervention, mental health, behavioral health, mobile app

## Abstract

**Background:**

eHealth interventions have proven to be valuable resources for users with diverse mental and behavioral health concerns. As these technologies continue to proliferate, both academic researchers and commercial app creators are leveraging the use of features that foster a sense of social connection on these digital platforms. Yet, the literature often insufficiently represents the functionality of these key social features, resulting in a lack of understanding of how they are being implemented.

**Objective:**

This study aimed to conduct a methodical review of commercially available eHealth apps to establish the SoCAP (social communication, affiliation, and presence) taxonomy of social features in eHealth apps. Our goal was to examine what types of social features are being used in eHealth apps and how they are implemented.

**Methods:**

A scoping review of commercially available eHealth apps was conducted to develop a taxonomy of social features. First, a shortlist of the 20 highest-rated eHealth apps was derived from One Mind PsyberGuide, a nonprofit organization with trained researchers who rate apps based on their (1) credibility, (2) user experience, and (3) transparency. Next, both mobile- and web-based versions of each app were double-coded by 2 trained raters to derive a list of social features. Subsequently, the social features were organized by category and tested on other apps to ensure their completeness.

**Results:**

Four main categories of social features emerged: (1) communication features (videoconferencing, discussion boards, etc), (2) social presence features (chatbots, reminders, etc), (3) affiliation and identity features (avatars, profiles, etc), and (4) other social integrations (social network and other app integrations). Our review shows that eHealth apps frequently use resource-intensive interactions (eg, videoconferencing with a clinician and phone calls from a facilitator), which may be helpful for participants with high support needs. Furthermore, among commercially available eHealth apps, there is a strong reliance on automated features (eg, avatars, personalized multimedia, and tailored content) that enhance a sense of social presence without requiring a high level of input from a clinician or staff member.

**Conclusions:**

The SoCAP taxonomy includes a comprehensive list of social features and brief descriptions of how these features work. This classification system will provide academic and commercial eHealth app creators with an understanding of the various social features that are commonly implemented, which will allow them to apply these features to enhance their own apps. Future research may include comparing the synergistic effects of various combinations of these social features.

## Introduction

### Background

Research on eHealth interventions has demonstrated that technology-driven resources such as apps and web-based platforms can provide effective and easily accessible support to address both mental and behavioral health concerns [1-3]. These technologies have been woven into our daily lives, with the average person spending an estimated 3.5 hours a day on their cell phones [4], which counts toward a daily total of 6.5 hours online [5], as the demand for mobile- and web-based interventions continues to grow. A recent estimate suggests that there may be upwards of 350,000 eHealth apps available for download across app stores [6], with the current number expected to be significantly higher given the growing popularity of such apps in recent years.

However, the potential of these digital health interventions is beset by a lack of engagement among users [3,7-9]. A recent review of 10 self-guided eHealth interventions found that up to 79% of participants did not meet the criteria for minimal use (ie, completing 1 module or assessment or using the intervention at least once), with an average dropout rate of 43% [10]. As a result, improving participant engagement is a vital consideration in the implementation of such technologies.

Several eHealth implementation theories have pointed to the importance of social connection in enhancing adoption and engagement [11]. The normalization process theory framework suggests that the way users interact online can foster the development of new social norms that encourage the utilization of eHealth platforms [12]. Actor-network theory suggests that individual users act on their sense of agency (ie, their ability to choose how to engage virtually) and interact with both non-human (eg, user interfaces, multimedia, and content) and human components (eg, other users and moderators) of a system, which thereby creates meaning and coherence through such ongoing social interactions [13]. Therefore, eHealth technologies with feature sets that cultivate these social processes may enhance participant engagement.

Currently, behavioral or psychological eHealth interventions use various social features to engage their users. The literature on eHealth interventions documents a broad range of features.

### Synchronous Communication Features

Real-time communication mediums (ie, voice and video calls, “live” chats, etc) are widely used in eHealth interventions to connect participants with peers, facilitators, and clinicians. An example used videoconferencing to enhance social interaction for socially isolated older adults, by having a trained facilitator meet with participants for 4 sessions a week over the course of 6 months [14]. Videoconferencing interventions may also use a group format, which is structured similarly to in-person support groups. For example, the TeleGAIN program comprised a 12-week videoconferencing group intervention for individuals with aphasia that was led by speech-language pathologists [15].

### Asynchronous Communication Features

In contrast, asynchronous communication features, such as discussion boards or forums, allow for interaction among participants even if they are not present online at the same time [16,17]. For example, one study used a discussion board and recruited individuals with a history of mental disorders to moderate and offer peer support. This method encouraged participants to contribute to the discussion boards and to receive support from moderators who had the lived experience of coping with similar challenges [18]. Such features may be particularly helpful for connecting participants who are dispersed across different time zones or who may not have the ability to commit to a fixed meeting schedule.

### Social Presence Features

Other types of social features do not require the need for direct communication, while enhancing participants’ sense of *social presence*, defined as the perception that there are other individuals present in a digital environment [19-21]. Additionally, emerging research is being conducted on text- or voice-based conversational agents (ie, chatbots), which can deliver intervention content through language-based user interaction [22-25].

### Affiliation and Identity Features

Other features enhance a participant’s ability to represent themselves or affirm their affiliations with others, setting the stage for more meaningful online or offline interactions. For example, the use of *avatars* may enhance a sense of identity and affiliation. In a study of a digital dyadic intervention for family members of veterans with posttraumatic stress disorder, the platform allowed a nominated family member participant to interact with a customized avatar that represented their veteran [26]. Therefore, these virtual representations of identity may help users perceive themselves and others in novel ways, leading to increased engagement.

### Social Integrations

Additionally, researchers can leverage the broad user base and rich social feature sets of mature technologies like Instagram, Facebook, and other similar platforms through integration with their own platforms [27].

Taken together, the literature demonstrates how such social features can play a central role in eHealth interventions. The majority of the published research pertains to eHealth resources that have been developed by academic researchers from higher education or medical institutions, which may be distinguished from commercially available apps, which are typically profit-driven enterprises. The findings suggest a tendency toward resource-intensive social features that involve live communication among providers, clinicians, and peers. However, the cost of implementation should be weighed against the scalability of these interventions [28]. Hence, there may be an opportunity for interventions to leverage social features that enable automation or do not require immediate input from users.

Traditionally, eHealth research has been opaque in providing details about how such features are implemented since the focus of such applied work falls on the population of interest or the outcomes achieved. Therefore, a system of labeling and categorizing these social features will allow academic and commercial eHealth app creators to have a clearer understanding of the various social features that are commonly implemented, enabling them to apply these features in their own work.

This study aimed to establish the SoCAP (social communication, affiliation, and presence) taxonomy of social features by conducting a scoping review of commercially available eHealth apps. A scoping review was suitable for the purpose of the study since this method allows for flexibility in determining inclusion criteria and search methods, which we adapted for identifying and classifying a broad range of social features. First, we conducted a search of commercially available apps, and subsequently categorized the social features to derive our taxonomy. This may help researchers describe their work and draw comparisons between other eHealth platforms.

## Methods

### Overview

This study may be characterized by a scoping review, which is commonly applied to topics that are complex or have not been extensively reviewed before [29]. Conventionally, this methodology applies to a scope of literature as its focus. However, our method involved determining the variety and categories of social features through a review of commercially available apps. Through this process, we developed our taxonomy, identifying four main categories of social features: (1) communication features (asynchronous and synchronous), (2) social presence features, (3) identity and affiliation features, and (4) other social integrations.

To conduct the scoping review, the 6-step framework methodology of Arksey and O’Mayley [30] was adapted. This included a methodical search, screening, and qualitative synthesis of the findings. This process includes (1) identifying the research question; (2) identifying relevant eHealth and mHealth interventions; (3) selection; (4) charting the data; (5) collating, summarizing, and reporting results; and (6) finally, consultation with experts in the field. No protocol was registered prior to the commencement of the study.

### Eligibility and Screening

A shortlist of commercial eHealth and mHealth interventions was selected from One Mind PsyberGuide, a non-profit eHealth app review website maintained by researchers from the University of California, Irvine, and Northwestern University. The site is not affiliated with an industry and has a transparent review process. Each review is conducted by eHealth and mHealth specialists who have demonstrated expertise in the field, as opposed to anonymous reviews on commercial app stores, which may be paid, sponsored, or falsified.

### App Rating System

To derive a shortlist of eHealth and mHealth apps, the website’s app ratings were referenced, comprising their (1) credibility, (2) user experience, and (3) transparency by trained reviewers from the One Mind Psyberguide. Members of the study team were not involved in the rating process. At the time of writing, 207 apps were listed in the guide, with a subset of 30 apps fully reviewed and scored. In accordance with the guide’s rating system, *Credibility* ratings are made on a scale from 1.00 to 5.00 and include components like having a proposed goal, clinical input, research basis, etc. *User Experience* ratings are made on a scale from 1.00 to 5.00 and include components like engagement, functionality, aesthetics, etc. *Transparency* is rated as “acceptable,” “questionable,” or “unacceptable” and pertains to an app’s privacy policies and conforming to data storage and collection policies, with 1.00 representing the lowest rating and 5.00 representing the highest rating.

**Figure 1:**
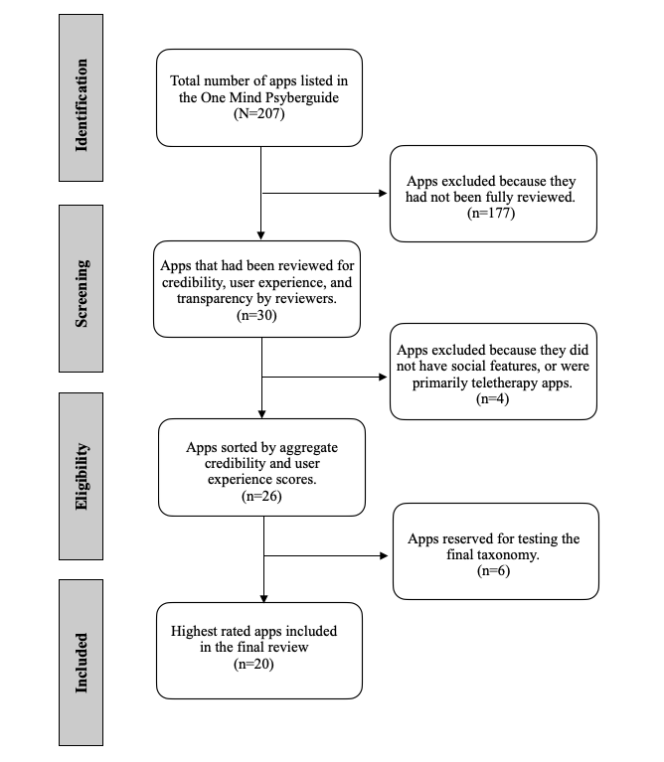
Flow diagram of the app search selection according to PRISMA (preferred reporting items for systematic reviews and meta-analyses) for scoping reviews.

Based on these ratings, only apps with “acceptable” credibility were selected, and the top 20 apps with the highest aggregate credibility and user experience were shortlisted. Apps that primarily relied on teletherapy were excluded because it would not be possible to see how their social features worked if we met with a counselor or therapist, which was beyond the scope of the review. We also excluded apps that did not have social features. A significant limitation of selecting apps that have been reviewed by One Mind PsyberGuide is drawing from a smaller pool of apps that may not fully represent the scope of apps available. However, given that our team would not be able to review a large number of apps, we felt that this approach would allow us to evaluate apps that had met independent quality benchmarks. The app selection process is charted in Figure 1.

The 20 shortlisted apps, in descending order of aggregate scores, include Headspace [31], Happify [32], Calm [33], SuperBetter [34], CogniFit [35], Sanvello [36], Lumosity [37], MoodMission [38], Wysa [39], Woebot [40], MoodKit [41], GG OCD [42], Virtual Hope Box [43], Sinasprite [44], NOCD [45], MindDoc [46], Replika [47], Simple Habit [48], Shine [49], and 365 Gratitude [50].

### Codebook Development

The top 20 apps from One Mind PsyberGuide that met the inclusion criteria were selected. Then, 2 independent coders (IK and MF) used the apps and categorized them according to the types of social features they involved. The remaining 6 apps that met inclusion criteria were reserved to test the SoCAP taxonomy after it was finalized.

Our first step was to create a preliminary codebook by referring to an existing social media feature taxonomy that focused exclusively on peer-based features in eHealth interventions [51]. The taxonomy comprised several categories and their corresponding features. However, the existing list primarily focuses on *peer-to-peer* features (eg, peer SMS, leaderboard, and peer commenting), which does not fully capture the range of social features in eHealth apps. Hence, the list was expanded to include features that may facilitate interactions with clinicians or study team members. Additionally, the list was expanded to include those that may enhance a sense of social presence (eg, pre-recorded videos and automated messages), even if they did not require direct input from users. Next, we organized the list of social features into a hierarchical structure, with 4 main categories that were informed by the existing literature. The four categories are (1) communication features (asynchronous and synchronous), (2) social presence features, (3) identity and affiliation features, and (4) other social integrations.

### Coding Process

The preliminary codebook was initially tested on a set of 5 apps that were randomly selected from the set of 20 apps. The coding process required the coders to note either the presence or absence of features. Both coders first performed independent coding using the preliminary codebook, and then subsequently convened to resolve discrepancies. This was done by reviewing the app together and collaboratively arriving at a decision. Subsequently, the necessary changes were made to the codebook. Next, the actual coding took place, in which both mobile- and web-based versions of each app were double-coded by the 2 coders. A first set of 5 apps was selected, where both coders (1) independently coded the apps, (2) shared their coding results, and (3) resolved any discrepancies in their coding. The double-coding process ensured that each app was extensively tested and reviewed in both mobile app and web-based formats. The next 5 apps were then coded using the same process, which was repeated until all 20 apps were reviewed. The final version of the taxonomy was then tested on 6 apps that had been reviewed by the One Mind PsyberGuide to confirm that no salient categories or features had been overlooked. There were no changes made to the codebook during this process.

As the coders reviewed the apps, specific use-cases that demonstrated innovation were identified. For example, this included (1) *novel* social interaction features, (2) existing features that have been *applied* in new ways for users, (3) features that may uniquely enhance a user’s sense of social presence, or (4) features that have not yet been addressed in the existing literature.

### Additional Analyses

In addition to noting the absence or presence of the social features, additional descriptive analyses were performed. This included the percentage of apps that addressed (1) psychological concerns (eg, symptoms of depression or anxiety and stress), (2) behavioral and health concerns (eg, exercise, diet, and smoking cessation), and (3) a combination of both psychological and behavioral and health concerns (eg, prenatal mental health). We also calculated the mean number of social features included per app.

## Results

### Overview

The SoCAP taxonomy consists of four main categories of social features: (1) communication features (asynchronous and synchronous), (2) social presence features, (3) affiliation and identity features, and (4) other social integrations. These categories and their corresponding descriptions are summarized in Table 1, while the specific features and their corresponding descriptions and examples are summarized in Table 2.

**Table 1:** Main categories of social media features.

Category	Description
Communication features	Features that allow for communication between 2 or more users; comprises (a) *synchronous communication features*, which allow for real-time communication, and (b) *asynchronous communication features*, which allow users to communicate without requiring them to be present at the same time
Social presence features	Features that enhance the perception of others on the platform
Affiliation and identity features	Features that enhance a participant's ability to represent themselves or affirm their affiliations with others
Social integration features	Features that leverage existing social networks or communication platforms

**Table 2:** The SoCAP (social communication, affiliation, and presence) taxonomy of social features.

Categories and features			Description and examples
**1. Communication features**			
	**(a) Synchronous features**		
		Chatrooms	Real-time discussion groups
		Voice calls	Voice-based calls via telephone, cell phone, Google Voice, etc
		SMS text messaging	Contacting other users, facilitators, or providers via text
		Videoconferencing	Video calls conducted in a group or individual setting
		Other	Other features that may be conducted in real-time; eg, live group meditations
	**(b) Asynchronous features**		
		Discussion boards	A bulletin board where users can interact by posting messages
		Email groups and mailing lists	A collection of users' email addresses, which allows them to send and receive emails from the group
		Expert support	Contacting a provider or facilitator through asynchronous means; eg, messaging and requesting support
		In-app peer messaging	Contacting other users directly by sending messages
		Other	Other features that may be conducted asynchronously; eg, commenting on videos
**2. Social presence features**			
		Chatbot	A feature that simulates human conversation through text or voice interactions
		Check-ins and surveys	Includes brief assessments and measures that collect user information
		Key quotes	Quotes from other users, thought leaders, etc
		Onboarding video and tutorial	Welcome messages or tutorials for users when they first join the platform
		Personal multimedia	Includes podcasts, videos, interviews, testimonials, recordings, etc
		Reminders and push notifications	Includes prompts, email reminders, notifications, etc
		Social data sharing and display	Displaying user activity data, feeds, or polls
		Social gamification	Game-like elements involving other users; eg, leaderboards and competitions
		Soliciting feedback	Questions or surveys that collect user feedback; eg, “How was this exercise?” and “Rate this lesson”
		Tailoring	Content or messages that are adapted based on user input; eg, personalized messaging and tailored information
		User counts	Displaying the number of participants who are engaging in online activities; eg, video views and number of users online
		Other	Other features that enhance social presence; eg, automated messages on user feeds
**3. Affiliation and identity features**			
		Accessibility panels and options	Includes settings to increase font size, narrate text, or enable voice commands to enhance accessibility
		Addressing participants by name	Includes personalized emails, notifications, welcome messages, etc
		Characters and avatars	Visual representations of other users, instructors, or characters
		Content for underserved groups	May include lessons, skills, activities, and other content for underserved groups; eg, track for anti-Asian hate and meditations for injustice
		Expert profiles	Individual profiles for facilitators, providers, and other experts.
		Network formation	Features that allow users to form networks; eg, befriending and following
		Peer groups and affiliation	Features that allow users to join groups based on their identity, interests, or concerns; eg, a discussion board for LGBTQ^a^ individuals and health care workers
		Personal avatar and photo upload	Users may be able to upload their own photos, customize avatars, or upload their own photos to present themselves to others
		Privacy and sharing options	Providing toggles for users to choose what content they would like to share or withhold from others
		Shared and family accounts	Allowing multiple users within one family account
		User profiles	A display of personal information about the user that may be public or private
		Other	Other affiliation and identity features
**4. Social integration features**			
		Social network integration	Interfacing with social networking sites such as Facebook and Instagram
		Other app integration	Interfacing with other apps such as WhatsApp and Viber
		Sending invites	Being able to invite others to join the app
		Other	Other social integrations and features

^a^LGBTQ: lesbian, gay, bisexual, transgender, and queer.

All (N=20) of the reviewed apps addressed psychological concerns, 85% (n=17) addressed behavioral and health concerns, and 85% (n=17) addressed a combination of both psychological and behavioral and health concerns (PRISMA [Preferred Reporting Items for Systematic Reviews and Meta-Analyses] flowchart shown in Figure 1). The mean number of social features included per app was 12.2 (SD 5.63, range 2-25).

### Communication Features: Asynchronous and Synchronous

**Figure 2:**
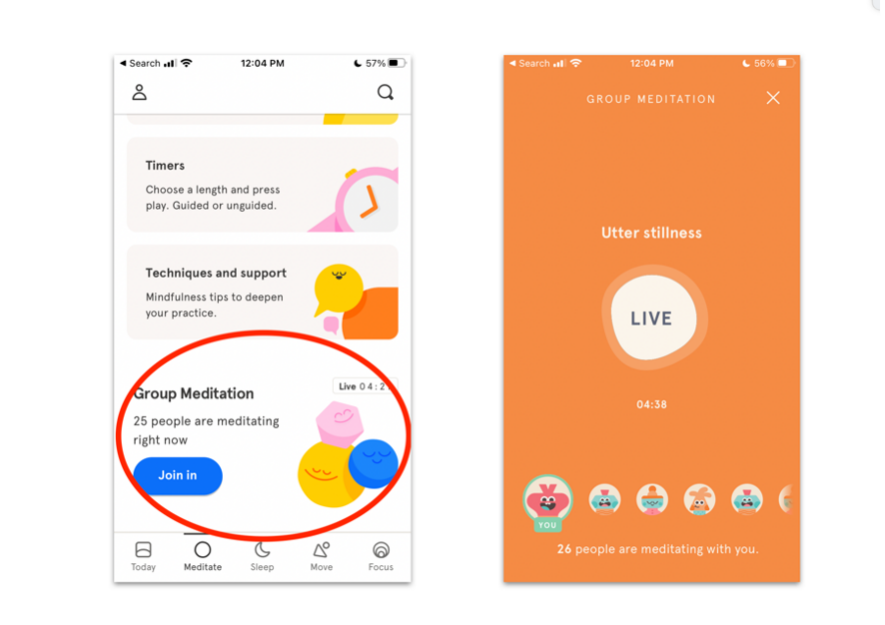
Synchronous features: “Live” meditation feature from Headspace.

Of the features identified in the taxonomy, those that facilitate synchronous (eg, texting and chatrooms) and asynchronous communication (eg, private messaging and discussion boards) provide the highest level of interaction with peers, facilitators, and providers. However, the boundary between “synchronous” and “asynchronous” features can be obscured depending on how these features are used. Therefore, the social features were categorized under one or the other based on their conventional use. An example of an innovative application of a synchronous feature was the “live” meditation feature from Headspace (Figure 2), in which users can follow along to a meditation that was led by a facilitator in real time. While participants are not able to directly interact with other participants, they are able to view how many others are engaging in the live activity, thereby fostering a sense of community among users.

A total of 25% (n=5) of the apps included at least 1 *synchronous* communication feature, with those apps including an average of 1.4 (SD 0.89, range 1-3) of such features. A total of 45% (n=9) included at least 1 *asynchronous* communication feature, with those apps including an average of 2.8 (SD 1.04, range 1-3) of such features. Taken together, 50% (n=10) of all apps did not include either an asynchronous or synchronous communication feature.

The reason for this may be that facilitating and moderating these communications may add an additional layer of complexity to maintaining these products. Each app averaged 0.35 (SD 0.75, range 0-3) synchronous and 0.7 (SD 1.08, range 0-3) asynchronous features; comparatively, our review found a *higher* average number of social presence features (mean 6.85, SD 2.32, range 2-10) and identity representation features per app (mean 4.40, SD 2.68, range 0-8), suggesting a heavy reliance on these automated features that require little or no direct input to enhance a sense of social connection. This may indicate developers’ preference for social features that are less resource-intensive.

### Social Presence Features

One construct that has been widely studied in online communities is the phenomenon of social presence and its effect on engagement and user satisfaction. This construct was initially adopted for e-learning research but has since been applied to various domains of research involving social interactions in digital spaces.

In our review, social presence features accounted for the most common type of social feature being implemented. 100% (n=20) of the apps included at least 1 social presence feature, with apps including an average of 6.85 (SD 2.32, range 2-10) of such features. These features appeared crucial in enhancing the sense that other users or staff member facilitators or providers were engaged with the app—without requiring a high level of human input. In general, all types of social features, including the previously described asynchronous and synchronous communication features, may enhance a sense of social presence. However, those that facilitate communication require a higher level of input and user interaction. Therefore, this separate category of social presence features was created, which enhances the perception of others without requiring as much input.

**Figure 3:**
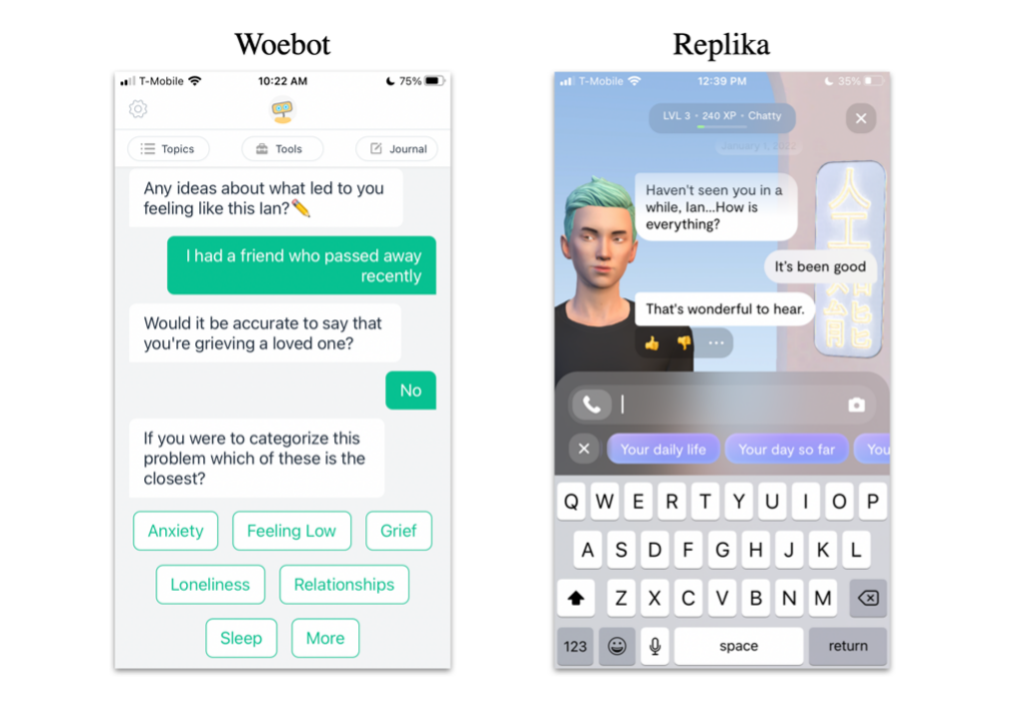
Social presence features: AI-driven chatbots featured in Woebot and Replika.

Some notable examples of social presence features include the artificial intelligence (AI)–driven chatbot featured in the Woebot app (Figure 3), which relies on conversing with a virtual robot character to access a wide range of psychological and behavioral health intervention content. Another instance where a chatbot is central to the user experience is the replica app, where users can interact with customized avatars.

**Figure 4:**
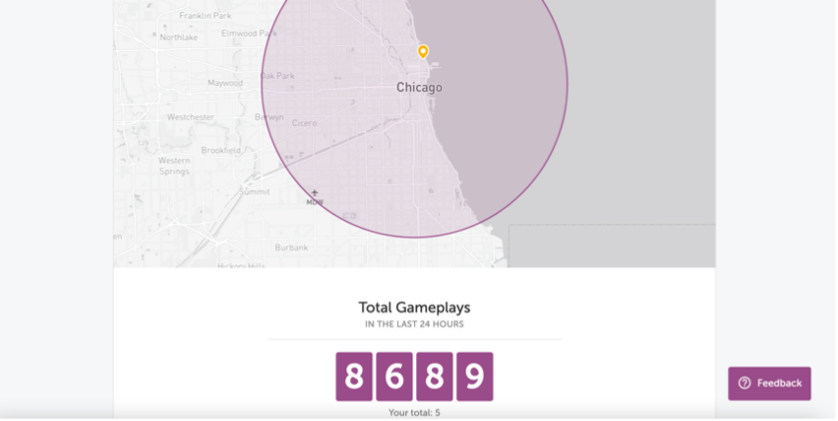
Social presence features: community map and user counts in the Lumosity app.

Another common social presence feature was the application of check-ins and surveys, which were deployed to gauge the participant’s mood, interest level, or perceived benefit of certain features or modules. The solicitation of user feedback bolsters the impression that a user’s inputs are valued and may enhance a general sense that their preferences are being acknowledged. This enhances the perception that there is an actual person “behind” the app, thereby fostering a sense of social presence. For example, in the posttraumatic stress disorder Coach app (Figure 4), users perform a brief check-in before and after exercises to indicate how they are feeling and to indicate how helpful they found the exercise.

Additional social presence features include user accounts, where apps may display how many people have engaged with certain content or might be on the platform at the same time. An example of this is the community map in the Lumosity app, where participants are shown a map of their location, and are able to view a count of how many people in the geographic region have logged on to the app or have engaged in similar activities as them recently.

### Affiliation and Identity Features

Through our review, the category of identity and affiliation features emerged. These are the features that do not require direct communication or interaction with others but provide the foundation necessary for such engagement. Through customization or personalization, these features allow users to present themselves to others in digital spaces. Such features may also reinforce group identities, allowing participants to experience a sense of affiliation and community [52,53]. This may be particularly important in fostering a sense of social connection in the context of eHealth apps, since participants may not have the ability to communicate with each other.

**Figure 5:**
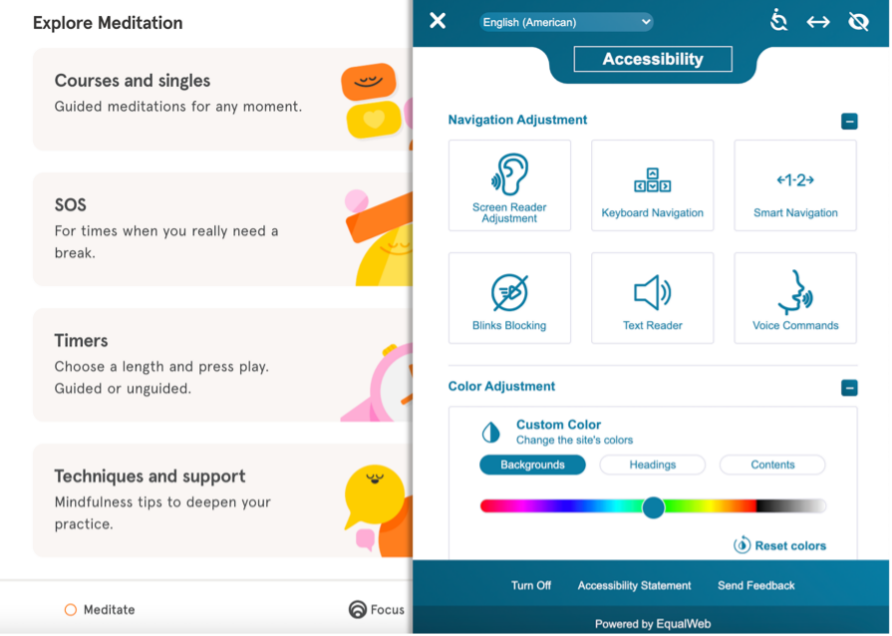
Affiliation and identity features: accessibility options in the Headspace app.

In total, 95% (n=19) of the apps included at least 1 identity and affiliation feature, with apps including an average of (mean 4.40, SD 2.68, range 0-8) of such features. The most common identity and affiliation features were user profiles, avatars, photo upload features, and addressing participants by name. Several apps also provide extensive accessibility options for individuals with different needs. One example is the Headspace app (Figure 5), which uses an EqualWeb toolbar to provide various options to make their website more accessible to users with sensory impairments or disabilities. These inclusive design features improve access, thereby allowing users with unique needs to fully use these eHealth apps. This enables users to participate on their own terms and affirms the presence of users with different support needs.

**Figure 6:**
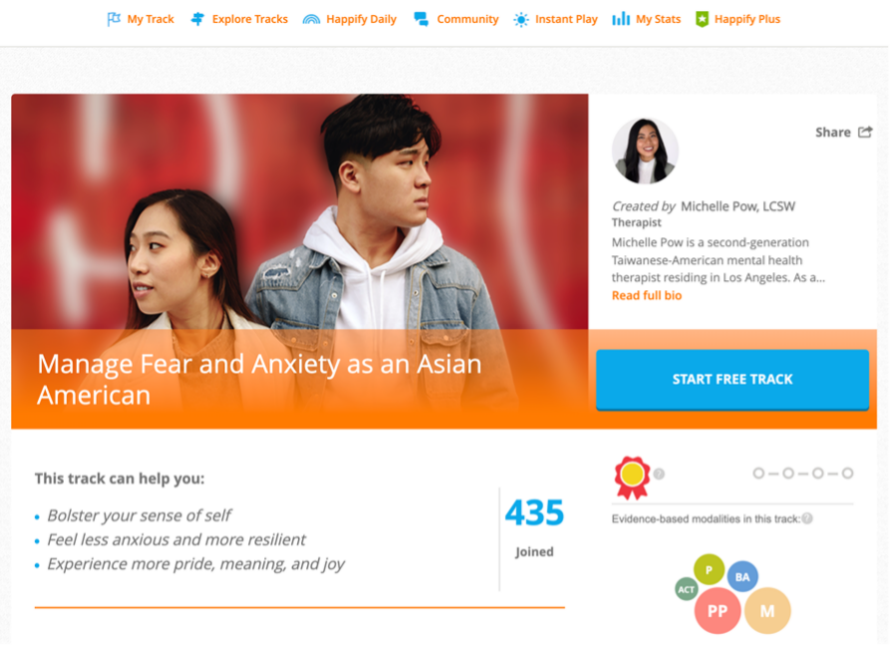
Affiliation and identity features: content for underserved user groups in Happify.

In the context of fostering affiliation, some features allowed users to form informal networks by following or befriending others, while other features allowed participants to opt into courses or online groups designed for specific populations (eg, expectant mothers and health care workers). Notably, several apps provide content for underserved groups who may be experiencing increased distress due to their racial or other identity. For example, Happify offers a track for Asian American users who may be experiencing racial discrimination (Figure 6). This was developed by a Taiwanese American therapist who teaches users how to manage feelings of fear and anxiety that have arisen from anti-Asian sentiment and also how to experience pride in their unique identities in spite of this adversity. Other examples include the Headspace app, which offers content addressing injustice and privilege, and the Wysa app, which provides support for LGBTQIA (lesbian, gay, bisexual, transgender, queer, intersex, and asexual) individuals. Taken together, such features help foster a sense of social connection by acknowledging the unique needs of these underserved user groups.

### Other Social Integrations and Features

It was found that most eHealth apps have features that leverage existing social networks or communication platforms. For example, the Shine app provides users with membership in a private Facebook group where they can participate in discussions. Another example is the Simple Habit Meditation app, which allows users to share their achievements on Facebook and Instagram. Other related features include allowing users to send email invites to refer others to join the app as members. Such social features maximize the use of existing social networks or communication technologies and may serve as channels to grow their user base. Additionally, such features may potentially enhance the interactivity of the apps, thereby increasing engagement. In total, 85% (n=17) of the apps included at least one form of social integration or other social features, with apps including an average of 2.35 (SD 1.00, range 0-7) of such integrations and features.

## Discussion

### Principal Findings

This study involved the development of the SoCAP taxonomy of social features through a scoping review of social features in commercial eHealth apps. To derive the taxonomy, a search of commercially available apps was first conducted. Subsequently, we categorized the social features to build the taxonomy.

It comprises four categories: (1) communication features (asynchronous and synchronous), (2) social presence features, (3) affiliation and identity features, and (4) other social integrations. It was found that social presence features and identity and affiliation features were more prevalent, encompassing a wider range of functionalities.

In contrast, our literature review found that social features currently used in research-driven eHealth apps tend to leverage synchronous interactions with peers, study staff, or providers; allowing them to meet the needs of high-acuity participants (eg, severe mental illness and patients with medically complex presentations). However, these social features are likely to require significant human resources to maintain since they are often reliant on real-time interactions. Through our initial literature review, it was found that the descriptions of how these features work were often minimal, given that the functionality of these eHealth platforms was typically secondary to the content being delivered.

In terms of social features in commercial eHealth apps, it is likely that there may be additional cost-driven constraints due to an emphasis on profitability. Our review found that commercially available apps lean heavily on automated features, which do not require as much human input as communication-heavy features, which require ongoing curation and moderation. While there are no definitive explanations for this, one hypothesis may be that most commercially available apps are typically profit-driven enterprises, and thus focus on the most cost-efficient use of human resources to deliver these eHealth products. Additionally, automated features may be more desirable for users in their ability to enhance social connection without others being present on the platform at the same time or to enhance their sense of privacy by not having to engage in direct communication. Therefore, researchers might look to commercial eHealth apps for insight on developing (1) social presence, (2) identity and affiliation, and (3) social integration features that enhance their participants’ sense of social connection in a way that is cost-efficient.

### Further Research and Implications

The proposed SoCAP taxonomy provides researchers with a framework to evaluate the range of social features currently being implemented in eHealth apps. This enables those who are less familiar with the technical aspects of eHealth intervention design to understand how these features might work and to describe the functionality of the eHealth interventions that they are implementing. The current taxonomy also enables greater transparency about the user-experience design and technical aspects of how eHealth apps may work by establishing common technical terms that both scientists and technologists can use. This fosters a greater understanding of the social technologies that are being applied in both research and industry.

### Strengths and Limitations

This study incorporates insight from both academia and industry. This involved combining an overview of social features in the eHealth literature, with a hands-on review of social features in commercially available eHealth apps. Since few of the interventions described in the literature are available for third-party access, the scoping review of commercially available apps allowed us to systematically test and use the apps in order to develop our taxonomy while drawing from the trends and insights gained from the narrative summary of the existing eHealth literature.

However, our scoping review has several limitations. First, it was conducted on a limited set of eHealth apps. This limitation was mitigated by testing the taxonomy on other commercially available apps. However, it remains to be proven how generalizable the taxonomy will be to academic research. Therefore, further research on the taxonomy could include its application to a broader set of eHealth apps in academic research. This may be performed through a conventional systematic literature review or conducted more extensively by contacting researchers directly to gain permission to use and evaluate their interventions. This would allow for further refinement of the taxonomy.

Second, another limitation relates to the nature of the rapidly evolving eHealth landscape; in which new operating systems, data transmission, and mobile technologies are constantly evolving. Therefore, a shortcoming of this screening method is that apps that may have recently been made available might not be included in this list.

This will result in some of the individual social features identified being obsolete over time, with emerging features that should be added to the SoCAP taxonomy. Yet, the broader categories of (1) communication features (asynchronous and synchronous), (2) social presence features, (3) affiliation and identity features, and (4) other social integrations are likely to be flexible enough to include more recent additions to the field. Nonetheless, the taxonomy should continue to be updated to account for these developments and mitigate obsolescence.

### Future Directions

Given that social presence and identity and affiliation features were frequently featured in the apps reviewed, we anticipate that there will be continued innovation in these categories. For example, refinements in AI algorithms are allowing chatbots to match users’ personalities in order to enhance engagement and satisfaction in these communications [54]. Another potential area of growth would be social features leveraging virtual reality, where users interact with immersive, digital environments and augmented reality technologies that integrate both simulated and real-life experiences. A notable example is Facebook’s ambitious efforts to develop virtual reality and augmented reality applications for its Metaverse initiatives. These are likely to result in the development of more immersive social features.

As these emerging social technologies are integrated into eHealth interventions, they will be accompanied by questions on how these innovations lead to measurable outcomes. In this study, we have developed an extensive list of social features. Future research may involve the application of the SoCAP taxonomy to evaluate different feature sets and compare their outcomes, for example, effectiveness and user preference. The growing complexity of eHealth technologies also necessitates an understanding of how an expanding social feature set might burden users—and, on the flip side, establishing a “baseline” amount of social features that could still efficiently enhance social connection. Taken together, the SoCAP taxonomy may provide app developers with a reference for categorizing and evaluating these features.

### Conclusions

This study involves the development of SoCAP, a taxonomy of social features in eHealth interventions. Through our scoping review of social features in commercially available eHealth apps, we identified four main categories in the SoCAP taxonomy; these include (1) communication features, (2) social presence features, (3) identity and affiliation features, and (4) other social integrations and features. The taxonomy represents a pioneering initiative to provide researchers with a shared understanding of the scope of social features currently being implemented in eHealth apps. This will allow researchers to apply these features to their own work, thereby fostering further innovation. This study found a heavy reliance on automated features that establish a context for meaningful interactions among users. We anticipate significant innovation in automated features in the future, particularly with the maturation of AI technologies. However, future research may involve further validating the SoCAP taxonomy across a broader range of research-oriented and commercially available apps and identifying which may be linked with enhanced user engagement and outcomes. The continued application and refinement of the SoCAP taxonomy will allow it to evolve as a robust tool for eHealth app development.

## Appendix

Multimedia Appendix 1 PRISMA checklist.

## Abbreviations

AI: artificial intelligence
LGBTQIA: lesbian, gay, bisexual, transgender, queer, intersex, and asexual
PRISMA: preferred reporting items for systematic reviews and meta-analyses
SoCAP: social communication, affiliation, and presence

## Data Availability

The data collected in the coding process may be obtained by contacting the first author.
